# Spatio-temporal dynamics of malaria in Rwanda between 2012 and 2022: a demography-specific analysis

**DOI:** 10.1186/s40249-024-01237-w

**Published:** 2024-09-16

**Authors:** Felix K. Rubuga, Paula Moraga, Ayman Ahmed, Emmanuel Siddig, Eric Remera, Giovenale Moirano, Guéladio Cissé, Jürg Utzinger

**Affiliations:** 1 Swiss Tropical and Public Health Institute, Allschwil, Switzerland; 2 University of Basel, Basel, Switzerland; 3 College of Medicine and Health Sciences, University of Rwanda, Kigali, Rwanda; 4 Computer, Electrical and Mathematical Sciences and Engineering Division, King Abdullah University of Science and Technology, Thuwal, Saudi Arabia; 5 Institute of Endemic Diseases, University of Khartoum, Khartoum, Sudan; 6 Department of Medical Microbiology and Infectious Diseases, Erasmus Medical Center, University Medical Center Rotterdam, Rotterdam, The Netherlands; 7 Faculty of Medical Laboratory Sciences, University of Khartoum, Khartoum, Sudan; 8 Rwanda Biomedical Center, Kigali, Rwanda; 9 Department of Medical Science, University of Turin, Torino, Italy; 10 Barcelona Supercomputing Center, Barcelona, Spain; 11 Center for Impact, Innovation and Capacity building for Health Information systems and Nutrition (CIIC-HIN), Kigali, Rwanda

**Keywords:** Malaria transmission, Epidemiology, Public health, Rwanda, Spatio-temporal analysis, Rwanda

## Abstract

**Background:**

Despite global efforts to reduce and eventually interrupt malaria transmission, the disease remains a pressing public health problem, especially in sub-Saharan Africa. This study presents a detailed spatio-temporal analysis of malaria transmission in Rwanda from 2012 to 2022. The main objective was to gain insights into the evolving patterns of malaria and to inform and tailor effective public health strategies.

**Methods:**

The study used yearly aggregated data of malaria cases from the Rwanda health management information system. We employed a multifaceted analytical approach, including descriptive statistics and spatio-temporal analysis across three demographic groups: children under the age of 5 years, and males and females above 5 years. Bayesian spatially explicit models and spatio scan statistics were utilised to examine geographic and temporal patterns of relative risks and to identify clusters of malaria transmission.

**Results:**

We observed a significant increase in malaria cases from 2014 to 2018, peaking in 2016 for males and females aged above 5 years with counts of 98,645 and 116,627, respectively and in 2018 for under 5-year-old children with 84,440 cases with notable geographic disparities. Districts like Kamonyi (Southern Province), Ngoma, Kayonza and Bugesera (Eastern Province) exhibited high burdens, possibly influenced by factors such as climate, vector control practices, and cross-border dynamics. Bayesian spatially explicit modeling revealed elevated relative risks in numerous districts, underscoring the heterogeneity of malaria transmission in these districts, and thus contributing to an overall rising trend in malaria cases until 2018, followed by a subsequent decline. Our findings emphasize that the heterogeneity of malaria transmission is potentially driven by ecologic, socioeconomic, and behavioural factors.

**Conclusions:**

The study underscores the complexity of malaria transmission in Rwanda and calls for climate adaptive, gender-, age- and district-specific strategies in the national malaria control program. The emergence of both artemisinin and pyrethoids resistance and persistent high transmission in some districts necessitates continuous monitoring and innovative, data-driven approaches for effective and sustainable malaria control.

**Graphical Abstract:**

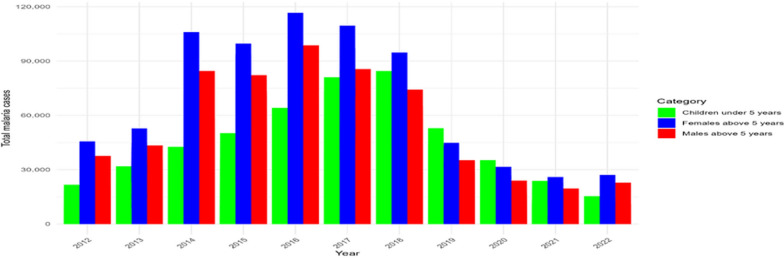

## Background

Malaria is a life-threatening disease caused by *Plasmodium* parasites that are transmitted through the bites of infected female *Anopheles* mosquitoes. Malaria remains a public health challenge and is a major focus of the Sustainable Development Goals (SDGs) [1]. Concerted global efforts resulted in a decrease in malaria incidence and mortality rates by 37% and 60%, respectively, from 2000 to 2015; yet, malaria continues to pose a considerable burden, especially in sub-Saharan Africa [2].

The World Malaria Report 2023, put forth by the World Health Organization (WHO), reported an increase in malaria cases with up to 249 million cases estimated in 85 malaria endemic countries and territories [3]. Notably, over 95% of these cases were attributed to 29 countries, with Nigeria (27%), the Democratic Republic of the Congo (12%), Uganda (5%), and Mozambique (4%) contributing most importantly to the global burden of malaria [3]. Furthermore, the report estimated over 600,000 deaths related to malaria in 2023, with more than 75% of these deaths occurring among children under the age of 5 years [3]. The burden can be attributed to various factors, including limited access to healthcare services, inadequate infrastructure, poverty, political instability, and environmental conditions that favor malaria transmission [4–6]. Additionally, challenges such as parasites resistant to antimalarial drugs, mosquitoes resistant to insecticides, and limited resources for prevention, control, and elimination further contribute to the persistence of malaria in this region [7].

In Rwanda, the burden of malaria was particularly severe at the beginning of the new millennium, marking the country one of the most affected in the world. At the time, health facilities across Rwanda reported over 5 million malaria cases. However, from 2005 to 2012, there was a reduction of approximately 86% in malaria incidence and 74% in malaria mortality, indicating substantial progress in controlling the disease. Despite these achievements, the dynamics of malaria transmission in Rwanda continue to evolve, particularly with differences between provinces, influenced by various factors, including demographic changes, climate variability, and public health interventions [8].

Despite the significant strides made in malaria control and elimination in Rwanda, there remains a critical need for ongoing, evidence-based strategic planning and interventions tailored to the evolving dynamics of malaria transmission in districts. This study fills an important gap in the current research by employing an up-to-date spatio-temporal analysis that incorporates detailed demographic and district-specific insights. Unlike previous studies that may have provided broad overviews of malaria incidence, our research specifically identifies which demographic groups and districts are at highest risk, enhancing the precision of public health interventions. By focusing on these key areas, this study not only enhances understanding of the current malaria landscape in Rwanda but also contributes to improving health equity in healthcare service delivery. We pinpoint underserved or high-risk groups and districts, facilitating the development and implementation of cost-effective interventions that are precisely targeted to meet the needs of these vulnerable populations.

The purpose of the current study was to analyse the spatio-temporal dynamics of malaria in Rwanda from 2012 to 2022, placing particular emphasis on demography-specific variations. The study sought to provide a comprehensive picture of the malaria situation in Rwanda, contributing to the national and global efforts in malaria control and elimination. Understanding the demography specific aspects of malaria transmission is crucial for effective disease management, as different population groups such as children, pregnant women, and the elderly exhibit varied susceptibilities to malaria [9]. These demographic distinctions play an important role in the overall dynamics of malaria transmission and its control. The findings from this study are expected to guide resource allocation and implementation of tailored malaria control strategies.

## Methods

In this study, a spatio-temporal analysis was conducted to investigate the patterns and dynamics of malaria transmission across Rwanda from January 2012 to December 2022. The analysis focused on identifying spatio-temporal trends and clusters of malaria transmission, providing insights into how these patterns have evolved over the years and contributing to a more nuanced public health strategy in Rwanda.

### Study setting

Rwanda is located in East Africa just south of the Equator, occupying an area of 26,338 km^2^. It is bordered by Uganda in the north, Tanzania in the East, the Democratic Republic of the Congo in the west, and Burundi in the south. The country is a part of the eastern and central African highlands, featuring a varied landscape that ranges from the mountainous Congo-Nile divide and Virunga volcano range in the West and North-Central areas to rolling hills, earning it the nickname “Land of a Thousand Hills” The average elevation across the country varies from 1500 to 2000 m above mean sea level.

Rwanda experiences a temperate, sub-equatorial climate with an average annual temperature of 18.5 °C. The mean annual precipitation is 1250 mm, concentrated in two main rainy seasons (March to May and September to November), interspersed with both a long (June to August) and a short dry season (December to February). An extensive network of rivers, streams, and lakes, surrounded by wetlands, characterizes the landscape of Rwanda.

According to the 2022 census, Rwanda had a population of 13,246,394 individuals, with a high density of 503 people per km^2^. The country is administratively divided into four provinces and the City of Kigali collectively encompassing 30 districts. The majority (72%) of Rwandans reside in rural areas and nearly half of the urban population lives in the capital city, Kigali. The demographic profile is predominantly young, with 70.3% of the population being under the age of 30 years.

Malaria transmission in Rwanda occurs year-round, peaking typically after the rainy seasons, in May/June and November/December. The entire population is at risk of malaria, though its transmission and intensity vary across regions due to factors like climate variability, altitude, population density, level of urbanisation, and population movement [10]. Malaria control measures in Rwanda include the distribution of long-lasting insecticidal nets (LLINs), indoor residual spraying (IRS), and larval source management (LSM). Environmental and climate factors, along with aspects like irrigation practices and cross-border movement, significantly influence the dynamics of malaria transmission in Rwanda [11].

### Data collection

This study utilized data from the Rwanda Health Management Information System (HMIS), which is not publicly available but can be accessed upon reasonable request. The dataset includes aggregated yearly data on malaria cases from 2012 to 2022, collected monthly for each of the 30 districts. through the national routine surveillance system from both community and health care facilities, and it are managed by the Rwanda Biomedical Center. Managed by the Rwanda Biomedical Center, the data are devoid of personal identifiers and categorize malaria cases into three demographic groups: children under the age of 5 years, males above 5 years, and females above 5 years.

Additionally, population data, crucial for calculating expected malaria cases and assessing relative risks (RRs), were obtained from the National Institute of Statistics of Rwanda. This information comes from the fourth (2012) and fifth (2022) Rwanda Population and Housing Census, thematic report on population size, structure, and distribution by district. These population figures were used to estimate the annual population for each district from 2013 to 2021, allowing for the analysis of malaria trends and demographic risks across districts [12, 13].

### Statistical analysis

**Fig. 1:**
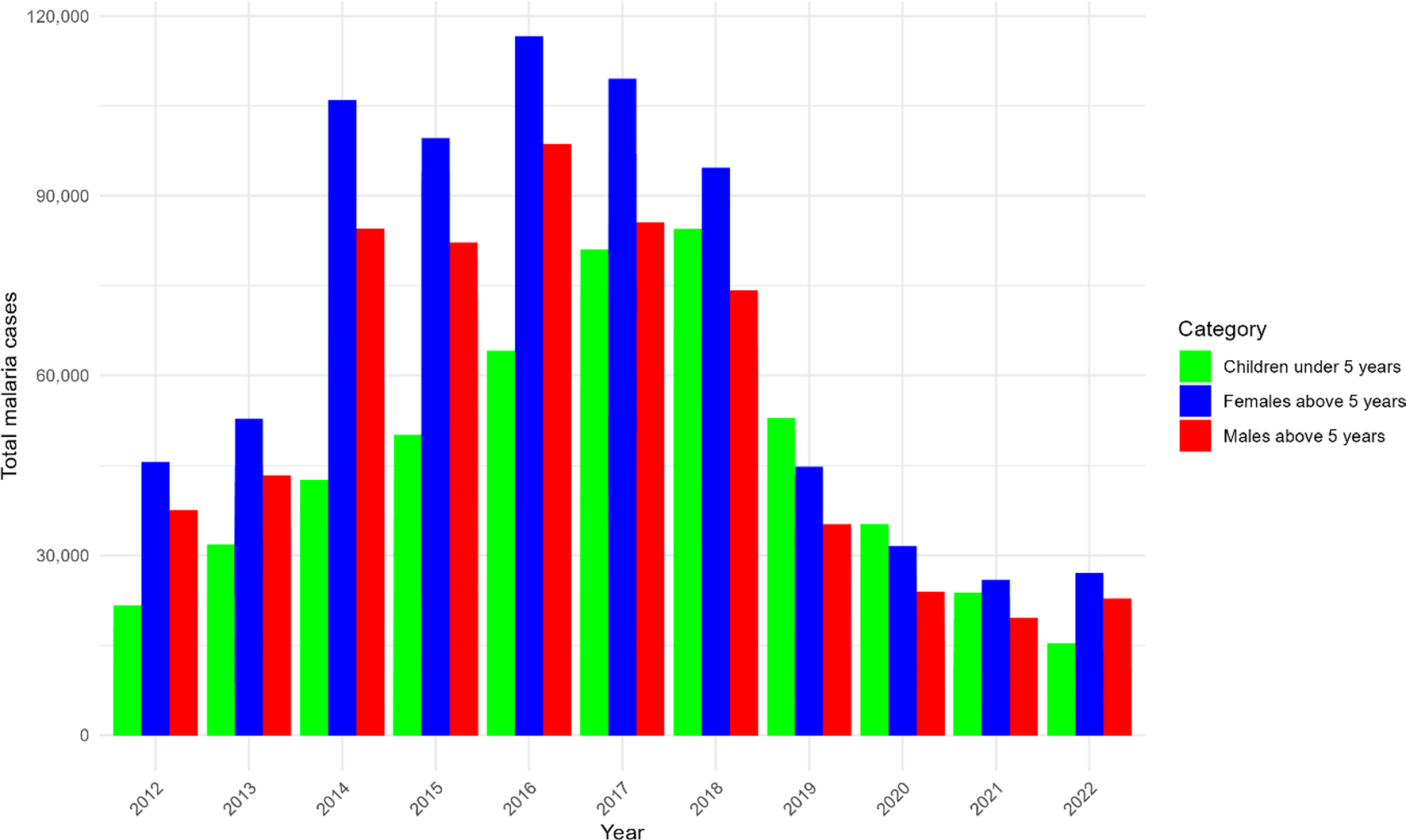
Annual trends in numbers of malaria cases, stratified by three demographic groups in Rwanda from 2012 to 2022

Initially, a descriptive statistics analysis encompassed bar plots for demographic groups; namely, children under the age of 5 years, males above 5 years, and females above 5 years. The bar plots in Fig. 1 provide a visual representation of the absolute number of reported malaria cases within the specified demographic groups from 2012 to 2022. Each bar color represents a different demographic group, illustrating the annual case counts and allowing for a comparative analysis over the years. This visualisation helps to identify trends and disparities in the incidence of malaria cases among these groups.

Subsequently, the analysis incorporated summary statistics, encompassing the median number of cases, standard deviation (SD), minimum and maximum for each demographic group across all districts. This step offered a quantitative insight into the variability and distribution of malaria occurrences.

For the spatio-temporal risk modeling and clusters of malaria in Rwanda, the first step focused on identifying and understanding the clusters of malaria cases across 30 districts of Rwanda, utilising SaTScan™ software version 10.1.2 (Martin Kulldorff, Information Management Services, Inc., Silver Spring, Maryland, USA). The analysis was performed using spatio scan statistics with the discrete Poisson model, a method particularly adept at identifying clusters with either significantly high or low rates of malaria incidence [14]. The discrete Poisson model was chosen to compare the observed number of cases against the expected distribution under the hypothesis of random spatiotemporal occurrence. This approach allowed for the identification of significant deviations from this expectation, thereby pinpointing areas and times of unusually high or low malaria prevalence. The spatially explicit analysis was conducted within defined circular windows, with the maximum spatio cluster size set at 50% of the population at risk. In the study, clusters were identified based on high or low rates. Additionally, the temporal window for the study ranged from a minimum of 1 year to a maximum of 5 years. A key aspect of the analysis was the rigorous statistical validation. There were 999 Monte Carlo replications to ascertain the statistical significance of the identified clusters, adhering strictly to a *P*-value threshold of 0.05 [15, 16].

In a second step, a spatio-temporal model was applied, using a Bayesian spatially explicit model, aiming to assess the RR of malaria infection across various districts and demographic groups in Rwanda from 2012 to 2022 [17, 18]. RR quantifies the likelihood of malaria occurrence in specific demographics (children under the age of 5 years, males above 5 years, and females above 5 years) across each district, compared to the national average.

In this context, the relative risk $\:{\theta\:}_{i}$ quantifies whether an area $\:i$ has higher $\:\left({\theta\:}_{i}>\:1\right)$ or lower $\:\left({\theta\:}_{i}<\:1\right)$ risk than the average risk in the whole population [19]. This measure helps identify high-risk zones, facilitating the implementation of targeted public health actions [20]. The observed malaria cases $\:{Y}_{ij}\:$ for district $\:i\:$ and year $\:j\:$ were assumed to follow a Poisson distribution:

$$\:{Y}_{ij}\sim\:Po\left({E}_{ij}{\theta\:}_{ij}\right),$$

where $\:{E}_{ij}$ represents the expected number of cases based on population data, and $\:{\theta\:}_{ij}\:$ denotes the RR [21]. The natural logarithm of the RR, $\:log\:\left({\theta\:}_{ij}\right)\:$ is expressed as a sum of an intercept and spatio-temporal effects, formulated as: $\:log\:\left({\theta\:}_{ij}\right)\:=\alpha\:+{f}_{BYM}\left(i\right)+{f}_{iid}\left(i,{t}_{j}\right)+{f}_{iid}\left(i,{t}_{j}^{2}\right)+\beta\:\times\:{t}_{j}+\:\delta\:\times\:{t}_{j}^{2}$. In this expression, $\:\alpha\:$ captures the baseline malaria risk, whilst $\:\beta\:$ and $\:\:\delta\:$ represent coefficients of the linear and quadratic terms of the temporal trend, $\:{t}_{j}\:$refers to the year index and ranges from 1 to the maximum number of years [18]. $\:{f}_{BYM}\left(i\right)\:$ represents the Besag-York-Mollié model for spatio structured and unstructured effects for location$\:\:i$; $\:{f}_{iid}\left(i,{t}_{j}\right)$ denotes the interaction between location and linear effect of time, assuming independent and identically distributed random effects for location $\:i$; $\:{f}_{iid}\left(i,{t}_{j}^{2}\right)$ captures the interaction between location and quadratic effect of time trends for location $\:\:i$. The spatio structure of the model was established by constructing a neighborhood matrix assuming as neighbors districts that share boundaries, effectively accounting for spatial dependencies [22]. This model’s capacity to handle the heterogeneity and variability in malaria risk due to geographic and temporal factors such as seasonal changes and shifts in public health strategies is enhanced by the integration of both spatial and temporal data. By capturing how malaria risk fluctuates over time in a particular district, the model aids in evaluating the effectiveness of interventions and supports evidence-based policy-making by delivering detailed insights into how risk varies among different demographics, districts, and over time, effectively guiding resource allocation and strategic public health planning [20]. Data segmentation into three demographic groups—children under 5 years of age, males above 5 years, and females above 5 years—was a critical step in the study. Post-model estimation, the estimated mean RRs and their 95% credible intervals were extracted. Inference was conducted using R-INLA methods [18]. Bayesian inference was conducted using integrated nested Laplace approximations (INLA) with the R-INLA package allowing for fast and accurate approximations of posterior distributions. To allow the empiric data to primarily influence the inference, vague priors were selected for our Bayesian analysis. These priors are intentionally non-informative, emphasizing the reliance on observed data to drive posterior distributions [23]. The statistical analyses and visualizations for this study were conducted using R version 4.0.3 (R Foundation for Statistical Computing, Vienna, Austria). Geographic data management and the creation of maps depicting the geographical spread of malaria risk over time were effectively handled using the *sf* package [24]. The *spdep* package was instrumental in analyzing spatial dependence and computing statistics that illuminate the spatial dynamics of the data [25]. Furthermore, the *ggplot2* package was utilized extensively to generate time plots, enabling a detailed observation of temporal trends in malaria risk from 2012 to 2022 [26–29].

## Results

### Descriptive analysis

From 2012 onwards, an increasing trend of malaria cases across all three demographic groups was observed. Cases among males and females older than 5 years peaked in 2016 with 98,645 and 116,627 cases, respectively (Fig. 1). Cases among children under the age of 5 years, kept increasing with an observed peak in 2018 with 84,440 cases.

For children under the age of 5 years, the median number of cases per district varied significantly, with some districts like Ngoma experiencing a median of 37,380 cases, illustrating a higher burden of malaria in these younger populations. The SD for this group indicates variability in case numbers, with districts like Kayonza and Ngoma (Eastern Province) showing a wide dispersion in the data (Table 1). The minimum and maximum for children under the age of 5 years further highlight the disparity between districts, ranging from as low as 23 in Nyabihu (Northern Province) to as high as 84,440 in Ngoma (Eastern Province) suggesting substantial heterogeneity in malaria burden among districts (Table 1).

For males above 5 years of age, the median cases also exhibited substantial variation, with districts like Nyabihu and Musanze (Northern Province) on the lower end, while others such as Kamonyi (Southern Province) and Bugesera (Eastern Province) showed higher medians (Table 1).

Similarly, for females above 5 years of age, the median cases by district revealed heterogeneity, with districts like Burera and Nyabihu on the lower spectrum, while others such as Kamonyi (Southern Province) and Bugesera (Eastern Province) had higher median numbers. The minimum and maximum in this demographic group underscore the heterogeneity in malaria burden across the country, with significant differences between districts’ lowest and highest recorded cases.

**Table 1:** Descriptive statistics of malaria cases distributed by age and sex demographics and districts of Rwanda between 2012 and 2022

District	Children under 5 years of age					Males aged above 5 years					Females aged above 5 years				
	Number of cases	Median cases	SD cases	Minimum cases	Maximum cases	Number of cases	Median cases	SD cases	Minimum cases	Maximum cases	Number of cases	Median cases	SD cases	Minimum cases	Maximum cases
Bugesera	299,879	22,154	18,441	9466	73,337	346,915	34,564	20,260	5610	75,848	434,820	41,441	26,071	6591	96,982
Burera	3419	220	277	100	1039	29,500	2141	1556	1140	6266	10,872	724	811	273	3078
Gakenke	19,804	1944	995	94	3703	70,025	4179	5068	704	18,132	61,386	3448	4719	295	16,721
Gasabo	160,182	15,336	8652	3955	31,700	294,080	22,836	19,078	8223	71,471	330,821	27,051	21,429	8007	78,694
Gatsibo	143,898	8267	13,218	1761	38,436	278,888	11,275	30,700	2032	85,075	335,816	14,089	36,926	2535	101,189
Gicumbi	28,837	2729	2158	199	6156	90,602	6976	7956	1304	29,337	95,934	8653	8126	1189	30,047
Gisagara	272,867	27,330	9092	9027	36,589	298,112	21,511	13,296	7716	48,081	363,296	25,957	15,799	9695	58,300
Huye	206,192	13,573	16,484	3357	56,249	372,226	27,069	32,460	3161	98,645	449,400	34,261	38,028	4546	116,253
Kamonyi	185,742	15,837	9496	1310	34,816	356,484	34,647	20,480	2576	65,114	448,618	44,816	25,357	3168	81,555
Karongi	71,641	6603	5049	274	15,957	169,582	10,645	15,384	748	50,451	193,985	10,462	17,920	687	58,313
Kayonza	342,883	31,077	24,126	2226	67,752	404,316	19,702	32,435	676	96,435	492,840	25,817	39,171	888	116,627
Kicukiro	58,489	4756	3515	421	10,038	111,451	8082	7601	1007	24,544	109,108	7969	7403	1042	23,656
Kirehe	159,202	8935	14,133	2390	42,886	276,622	14,875	28,853	2062	84,505	338,566	17,448	36,878	1936	105,984
Muhanga	86,804	9412	4740	257	16,129	202,912	14,362	16,722	678	54,028	235,811	17,194	19,086	660	60,989
Musanze	3920	333	259	49	726	24,018	1824	1581	633	6093	15,179	1158	1163	190	4366
Ngoma	393,286	37,380	31,582	555	84,440	452,820	24,597	35,409	251	89,943	575,928	32,918	45,199	292	116,496
Ngororero	25,358	2372	1595	37	5847	70,505	3529	6613	311	23,386	75,362	4160	7707	112	27,300
Nyabihu	1216	118	65	23	255	14,572	833	1230	308	4521	8230	402	841	148	3064
Nyagatare	150,183	9196	10,042	3386	34,894	219,499	11,217	19,215	1116	56,865	274,134	13,798	24,454	1394	72,046
Nyamagabe	78,319	7534	3992	170	12,984	155,988	11,636	11,407	574	40,886	190,408	14,017	13,956	489	50,381
Nyamasheke	172,147	11,533	12,047	1351	35,249	286,196	16,578	25,727	2598	79,468	337,510	19,728	30,513	2805	94,844
Nyanza	198,717	17,107	11,675	3674	34,833	367,842	27,398	25,236	2058	72,796	467,216	32,605	31,951	2705	91,525
Nyarugenge	45,828	3761	2800	268	7899	105,176	8049	7564	710	23,785	102,947	6958	7654	578	22,658
Nyaruguru	93,261	8549	5361	885	19,079	156,112	9687	12,393	1324	46,142	181,856	12,268	13,766	1477	51,265
Rubavu	30,987	2402	2616	67	8826	57,787	2233	7633	158	26,710	62,161	2422	8280	160	28,952
Ruhango	211,010	14,806	15,617	690	43,223	368,146	29,596	27,335	1017	70,181	444,359	34,513	32,987	1337	85,656
Rulindo	37,130	2944	2358	706	7737	99,638	5220	8425	1622	30,673	101,578	5498	8691	1433	31,614
Rusizi	182,699	10,675	11,041	5053	33,934	217,693	11,484	18,242	2726	50,023	261,816	13,322	21,965	3376	59,784
Rutsiro	36,667	3329	2257	76	7019	90,191	5309	7457	371	25,729	84,663	5219	7158	313	25,369
Rwamagana	224,216	15,450	14,316	1975	42,270	329,962	27,621	22,426	1701	61,943	395,646	33,969	27,231	1803	74,099

Notes:

Number of cases = Total reported malaria cases per district for the years 2012–2022 by demographic group (children under 5, males above 5, females above 5)

Median cases = The median value of malaria cases per year for each district and demographic group

SD (Standard deviation) Cases = Measures the annual variability of malaria cases per district for each demographic group, showing how much case numbers fluctuate from the median

Minimum cases = The lowest annual malaria case count recorded from 2012–2022 for each district and demographic group, highlighting the minimum impact period

Maximum cases = The highest annual malaria case count recorded from 2012–2022 for each district and demographic group, indicating peak periods of impact

Aggregated malaria incidence rates per 1000 individuals for the period 2012–2022 are detailed across various districts, stratified by three demographic groups as shown in Table 2. The district of Ngoma recorded the highest incidence rate among children under the age of 5 years, with 670.5 cases per 1000 children. Conversely, the district of Nyabihu reported the lowest rate in this group, with 2.6 cases per 1000 children. Among males above 5 years of age, the highest incidence rate was observed in Ngoma with 273.7 cases per 1000, while Musanze had the lowest with 12.6 cases per 1000. Similarly, females above 5 years of age in Ngoma experienced the highest incidence rate of 313.9 cases per 1000 individuals, whereas Burera reported the lowest rate at 6.0 cases per 1000.

**Table 2:** Malaria incidence rates per 1000 individuals in Rwanda from 2012–2022 by district and demographic group

District	Children under the age of 5 years	Males above 5 years	Females above 5 years
Bugesera	390.4	163.4	204.1
Burera	6.6	18.0	6.0
Gakenke	41.7	44.09	33.98
Gasabo	150.7	86.0	101.2
Gatsibo	188.1	125.9	138.1
Gicumbi	48.7	46.9	45.2
Gisagara	463.7	189.6	202.2
Huye	403.2	225.3	257.9
Kamonyi	324.5	197.8	226.8
Karongi	139.7	103.3	108.1
Kayonza	538.4	222.9	252.0
Kicukiro	106.9	55.9	57.0
Kirehe	252.8	153.3	171.6
Muhanga	190.2	129.4	138.4
Musanze	6.5	12.6	7.1
Ngoma	670.51	273.7	313.9
Ngororero	47.9	46.1	41.9
Nyabihu	2.6	10.8	5.3
Nyagatare	164.1	86.1	101.8
Nyamagabe	156.2	96.9	105.1
Nyamasheke	271.2	159.8	163.6
Nyanza	386.9	231.0	277.1
Nyarugenge	101.9	63.1	68.3
Nyaruguru	199.1	114.1	118.7
Rubavu	39.7	26.8	27.1
Ruhango	429.6	239.6	260.3
Rulindo	81.5	68.1	61.8
Rusizi	254.6	108.9	121.3
Rutsiro	70.5	57.9	48.7
Rwamagana	371.6	175.1	207.9

Notes:

Children under the age of 5 years = This column shows the aggregated incidence rate of malaria per 1000 children under five years old in each district for the years 2012–2022

Males above 5 years = details the aggregated malaria incidence rate per 1000 males above the age of five in each district for the years 2012–2022

Females above 5 years = indicates the aggregated malaria incidence rate per 1000 females above the age of five in each district for the years 2012–2022

**Fig. 2:**
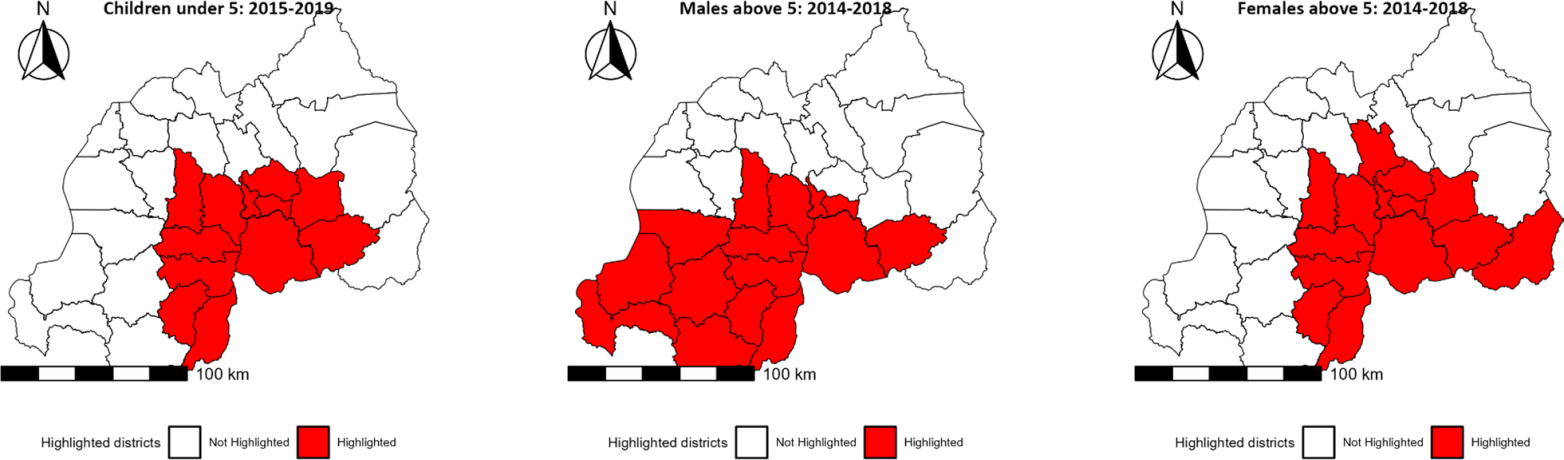
Spatial distribution of high malaria risk clusters in Rwanda from 2012–2022, stratified by demographic groups and their corresponding cluster identification periods. The left map indicates clusters for children under the aged of 5 years during 2015–2019, the middle map denotes clusters for males above 5 years of age during 2014–2018, and the right map illustrates clusters for females above 5 years of age during 2014–2018. Districts coloured in red represent areas where the number of observed malaria cases was higher than expected, signalling a higher risk of reported malaria cases compared to the national average

The spatio scan statistics highlighting the high-risk malaria clusters in Rwanda, stratified by demographic groups between 2012 and 2022 are depicted in Fig. 2. For children under the age of 5, the clusters identified during 2015–2019 include districts such as Bugesera, Gasabo, Gisagara among others, with an observed to expected case ratio of 2.29, indicating that the observed cases were over twice the expected number. This cluster also shows a significant log likelihood ratio of 584,362, strongly suggesting a higher-than-expected malaria incidence, with a *P*-value of less than 0.001, confirming the statistical significance of these findings. Similarly, for males above 5 years, the period 2014–2018 shows a cluster encompassing additional districts like Karongi and Nyamagabe, with an observed to expected ratio of 2.3 and a log likelihood ratio of 1,146,688, also significant at a *P*-value of less than 0.001. This indicates a similarly high risk compared to national averages, with significantly more cases observed than expected. The clusters for females above 5 years of age during the same period exhibit an observed to expected ratio of 2.39 and a log likelihood ratio of 1,469,933, with the clustering extending into districts such as Kirehe and Rulindo. The *P*-value of less than 0.001 for these clusters again supports the presence of a significantly higher incidence of malaria than expected based on the national average. These statistics substantiate the clusters shown in all maps, with districts marked in red indicating a number of observed malaria cases surpassing expected values.

A comprehensive summary of the spatio scan analysis from 2012 to 2022, depicted in Table 3 reveals malaria clusters across various demographic groups. The analysis indicates that for children under the age of 5 years, between 2015 and 2019, a total of 1,637,971 malaria cases were observed in districts including Bugesera, Gasabo, Gisagara among others. These cases notably surpassed the expected count of 713,780, based on the underlying population of 650,000. The expected number of cases is defined as the number of cases that would be expected based on the underlying population at risk in the absence of any spatial or temporal clustering. The high significance of this cluster is further emphasized by a log likelihood ratio of 584,362 and a *P*-value of less than 0.001. Similarly, for males aged above 5 years from 2014 to 2018, the observed cases totaled 3,032,237 across several districts, significantly exceeding the expected 1,318,150 cases derived from a population of 2,324,912. This discrepancy is highlighted by a log likelihood ratio of 1,146,688 and a *P*-value of less than 0.001. Additionally, the cluster for females aged above 5 years during the same timeframe involved 3,611,089 observed malaria cases, far exceeding the expected 1,510,183 cases from a population of 2,435,551. This clustering is confirmed as significant by a log likelihood ratio of 1,469,933 and a *P*-value of less than 0.001 (Table 3).

**Table 3:** Malaria cluster analysis summary for Rwanda in 2012–2022, stratified by demographic group

Demographic group	Cluster districts	Center latitude	Center longitude	Radius	Time frame start	Time frame end	Population	Total number of malaria cases	Total expected cases	Annual cases per 100,000	Observed/expected	Log likelihood ratio	*P*-value
Children under the age of 5 years	Bugesera, Gasabo, Gisagara, Huye, Kamonyi, Kicukiro, Muhanga, Ngoma, Nyanza, Nyarugenge, Ruhango, Rwamagana	−2.24	30.15	58.35	2015-01-01	2019-12-31	650,000	1,637,971	713,780	50,405	2.29	584,362	< 0.001
Males aged above 5 years	Bugesera, Gisagara, Huye, Kamonyi, Karongi, Kicukiro, Muhanga, Ngoma, Nyamagabe, Nyamasheke, Nyanza, Nyarugenge, Nyaruguru, Ruhango, Rusizi	−2.62	29.84	85.05	2014-01-01	2018-12-31	2,324,912	3,032,237	1,318,150	26,717	2.3	1,146,688	< 0.001
Females aged above 5 years	Bugesera, Gasabo, Gisagara, Huye, Kamonyi, Kicukiro, Kirehe, Muhanga, Ngoma, Nyanza, Nyarugenge, Ruhango, Rulindo, Rwamagana	−2.24	30.15	62.22	2014-01-01	2018-12-31	2,435,551	3,611,089	1,510,183	30,568	2.39	1,469,933	< 0.001

**Fig. 3:**
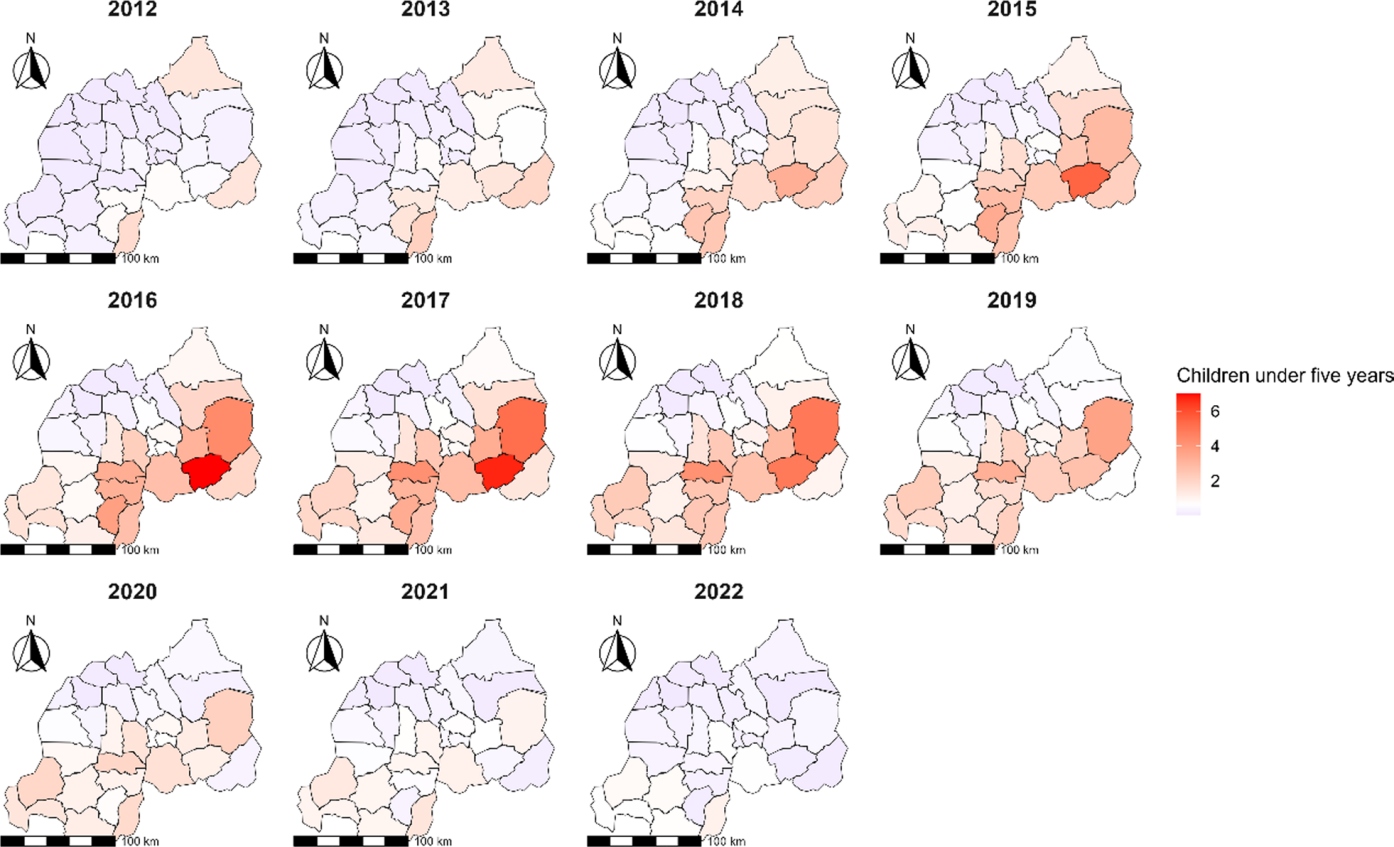
Map of Rwanda with spatio-temporal relative risks (RRs) of malaria for children under the age of 5 years from 2012 to 2022

Throughout the 2012 to 2022 timeframe, the spatio-temporal analysis of malaria risk among children under the age of 5 years has revealed distinct patterns of elevated RR in certain districts (Fig. 3). Initially, the district of Ngoma was notable with the highest RR in 2014, reaching 3.24 (95% *CI:* 3.22–3.26). Over the subsequent years, districts such as Gisagara, Huye, Kayonza, and Ruhango were consistently highlighted for their high RRs. Notably, Gisagara district exhibited a significant increase in RR, with a peak at 2.13 (95% *CI:* 2.12–2.15) in 2013 and surging at 2.64 (95% *CI:* 2.63–2.65) in 2015. Similarly, Huye district showed an increase in RR to 3.39 (95% *CI:* 3.38–3.41) in 2015. Ngoma district displayed the most substantial RR during this period, with a peak of 7.00 (95% *CI:* 6.98–7.03) in 2016 (Fig. 3).

**Fig. 4:**
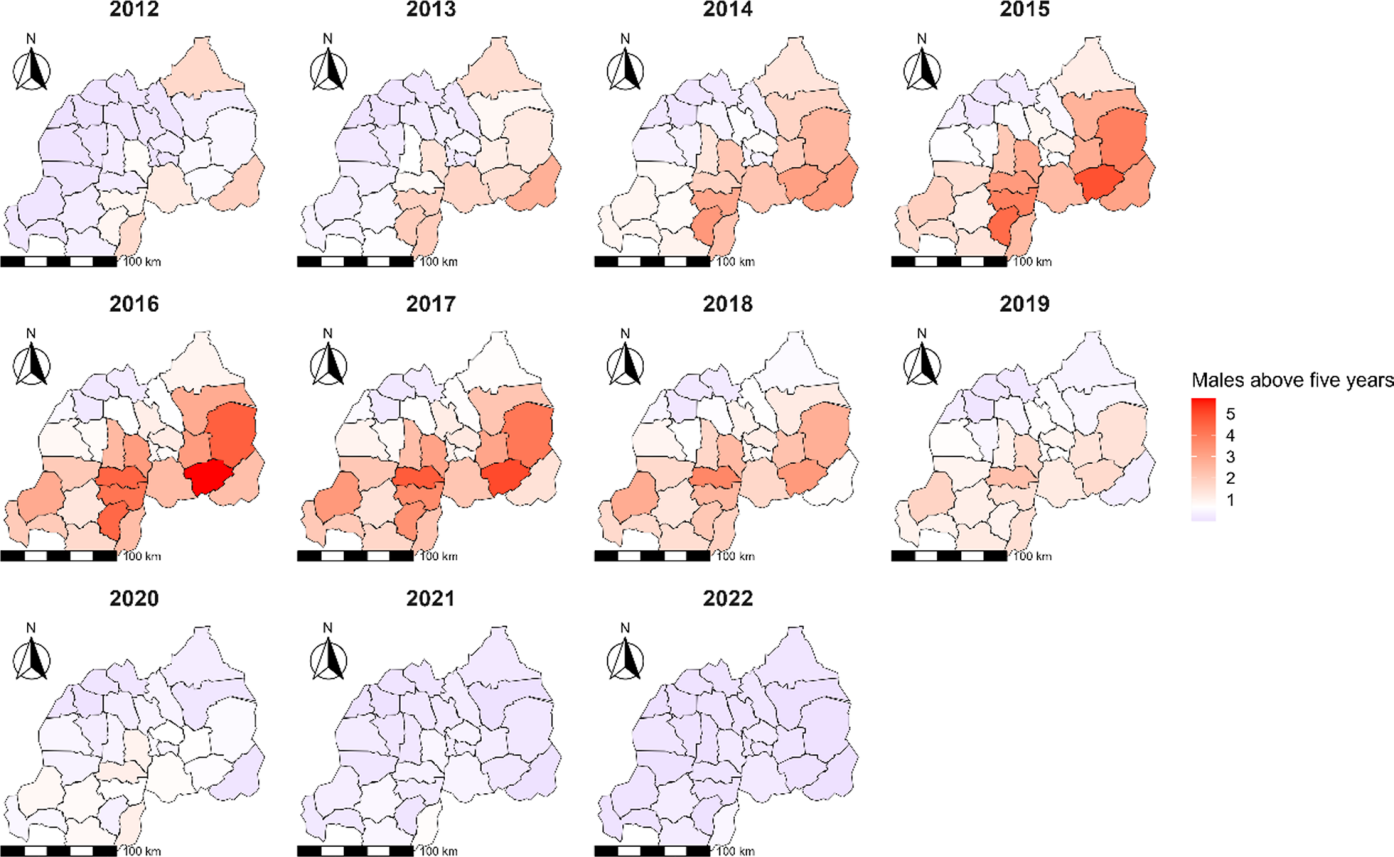
Map of Rwanda with spatio-temporal relative risks (RRs) of malaria for males aged above 5 years from 2012 to 2022

Over the decade-long study from 2012 to 2022, a discernible pattern of malaria risk among males aged above 5 years was observed, with significant variances across different districts. The investigation began with Kirehe district exhibiting a notable RR of 1.86 (95% *CI:* 1.84–1.87) in 2012 (Fig. 4). In subsequent years, several districts emerged as recurrent hotspots with elevated RR values. Ngoma district, for instance, showed elevated RRs throughout the study period, with a peak RR of 5.70 (95% *CI:* 5.68–5.72) in 2016. Similarly, Kayonza district displayed consistently high RRs, notably reaching 4.59 (95% *CI:* 4.57–4.60) in 2016. Ruhango district also demonstrated persistently high RRs, especially in 2017 with a RR of 4.67 (95% *CI:* 4.65–4.69). Despite a general trend of declining RRs by 2020, the districts of Ngoma, Kayonza, and Ruhango continued to report elevated risks. 

**Fig. 5:**
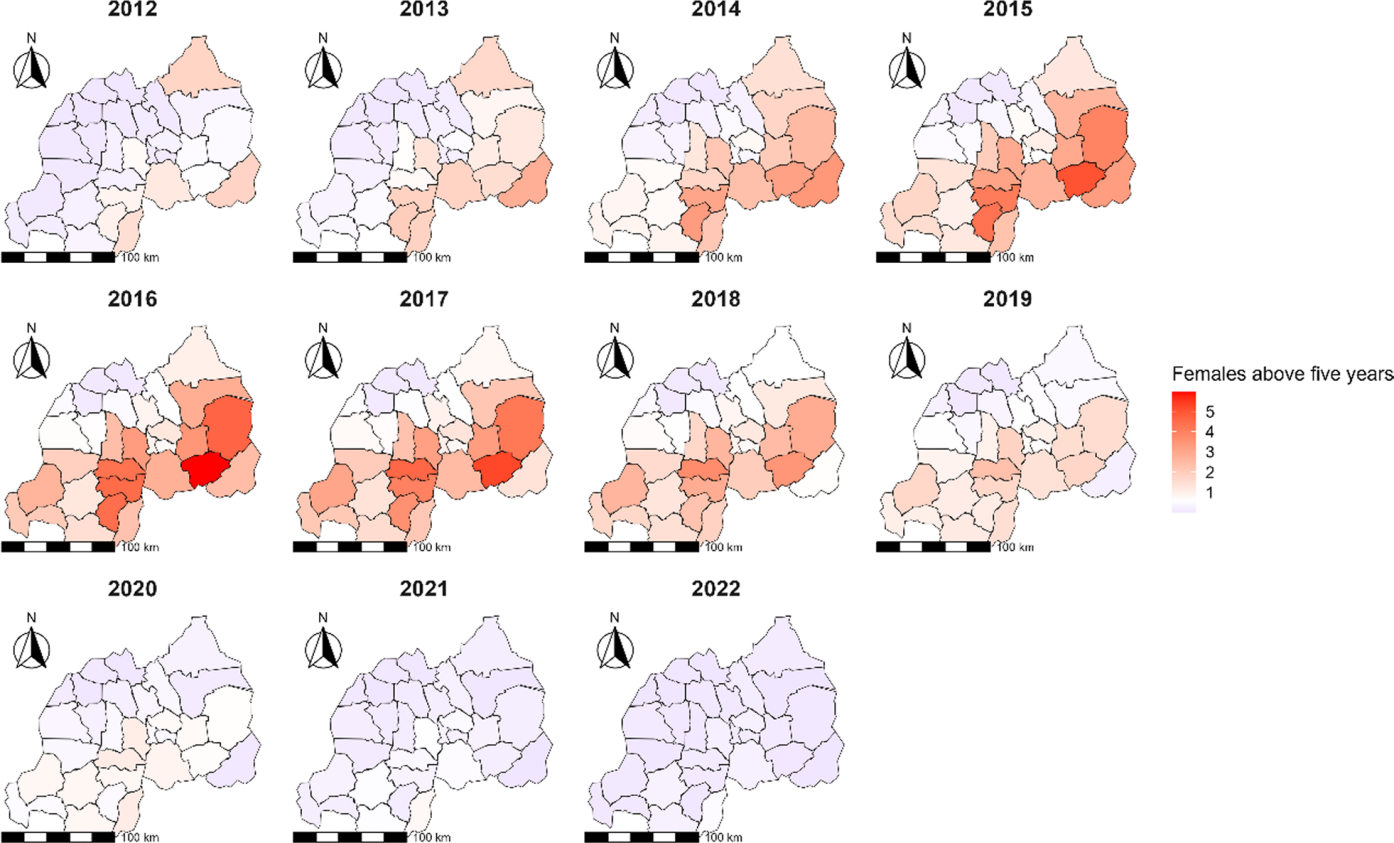
Map of Rwanda with spatio-temporal relative risks (RRs) of malaria for females aged above 5 years from 2012 to 2022

In the comprehensive analysis conducted from 2012 to 2022, notable trends in malaria risk among females aged above 5 years were observed, highlighting significant geographic heterogeneity in risk levels across different districts (Fig. 5). The investigation began with districts of Kirehe and Nyagatare which demonstrated elevated RRs of 1.77 (95% *CI:* 1.76–1.79) and 1.76 (95% *CI:* 1.75–1.78), respectively, in 2012. However, it was from 2014 onwards that certain districts consistently emerged with particularly high RRs (Fig. 5). In 2014, Kirehe district reported a markedly high RR of 3.45 (95% *CI:* 3.43–3.46), and Huye district also showed a substantial increase to 3.32 (95% *CI:* 3.30–3.33). The year 2015 saw Ngoma district reaching the decade peak with a RR of 5.04 (95% *CI:* 5.02–5.05), with Huye and Kayonza districts following closely, peaking at 4.35 (95% *CI:* 4.34–4.36) and 3.92 (95% *CI:* 3.91–3.94), respectively. Ruhango emerged as a district with an elevated malaria risk, peaking at a RR of 4.62 in 2017. Kayonza, Nyanza, and Nyamasheke were also identified as high-risk districts, consistently reporting RRs above 2.5 from 2014 to 2018 (Fig. 5). Toward the end of the study period, there was a general decline in RRs. However, Ruhango and Gisagara districts continued to manifest elevated risks of around 1.10 in 2020.

**Fig. 6:**
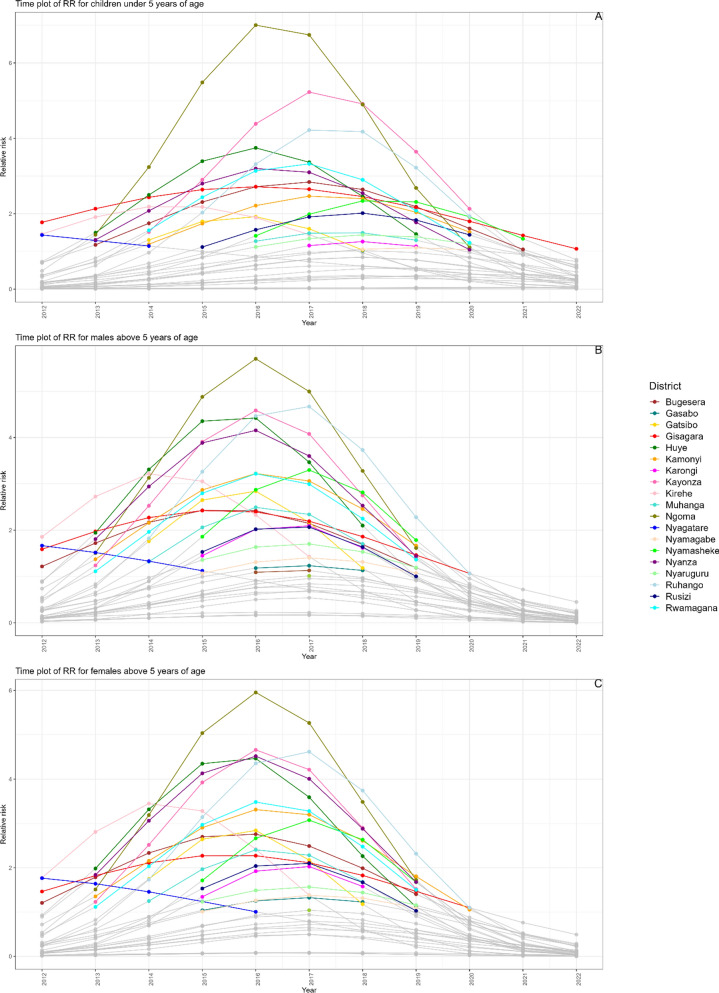
Combined time plots illustrating the relative risk (RR) of malaria infection for three demographic groups in districts with RR greater than 1 in Rwanda from 2012 to 2022. **A** Children under the age of 5 years; **B** males above 5 years of age; and **C** females above 5 years of age. Each colored line corresponds to a district that reported a RR greater than the national average during the study period. The color key denotes the specific districts

The time plots delineate the malaria transmission risk across various districts in Rwanda, stratified by demographic groups and annotated for clarity (Fig. 6). Plot A illustrates the trend for children under the age of 5 years, where districts such as Ngoma, Huye, Kayonza, and Ruhango exhibit an ascending and descending trajectory in RR from 2012 to 2022. In contrast, Gisagara and Bugesera districts demonstrate a relatively stable RR (Fig. 6).

Plot B, representing males above 5 years of age, highlights that Ngoma, Ruhango, Kayonza, and Huye districts faced a consistent increase in RR. The peak of the risk was observed in Ngoma district in 2016 (Fig. 6).

In Plot C, focusing on females above 5 years of age, districts such as Ngoma, Kayonza, Nyanza, and Huye are depicted with RRs of 2 and above (Fig. 6). In these plots, colors indicate RR values above 1 for some years in the study period, signifying a higher risk of malaria transmission. In contrast, those areas without color or unhighlighted represent districts with a RR of 1 or below.

### Interactive web application

An interactive web application has been created using Shiny (https://paulamoraga.shinyapps.io/malariarwandaapp/), The dashboard offers tools such as interactive maps, a database, heat maps, and risk analysis, enabling users to explore malaria data across Rwanda for various demographic groups from 2012 to 2022. It visualizes data like population, cases, and RR, providing insights into spatial and temporal trends to support public health planning and interventions [30].

## Discussion

The comprehensive spatio-temporal analysis of malaria risk in Rwanda from 2012 to 2022 provided important insights into the disease dynamics. The study focused on identifying spatial and temporal trends, as well as clusters of cases, unveiling the evolving patterns of malaria over an 11-year period. A significant increase in malaria cases was observed from 2014 to 2018, particularly affecting three demographic groups: children below the age of 5 years, and males and females aged above 5 years, with a peak in 2016. This surge was notably impactful on under 5-year-old children and females aged above 5 years. These observations concur with the 2015 Rwanda DHS report, which indicated an increased malaria prevalence among children under the age of 5 years, reflecting a broader trend of escalating cases [31]. Potential contributing factors to this rise included climatic variations that might favor mosquito breeding, as well as issues with LLINs, such as delayed deliveries and insufficient insecticide content [32]. Additionally, the confirmed resistance of *Anopheles gambiae* to pyrethroids and a shift in vectors toward outdoor biting, thus reducing the effectiveness of LLINs [32–34]. Moreover, lack of access to healthcare might play a role, including limited availability of effective antimalarial medications, insufficient staffing and training for healthcare workers, which might impact the management and containment of malaria and other infectious diseases. Challenges in funding and implementing prevention and control programs exacerbated by socioeconomic factors, including poverty, lack of awareness about malaria prevention, and limited access to education might have contributed. The decline in cases post-2018 might be attributed to the Rwandan government efforts in training community health workers (CHWs), which led to effective home-based malaria treatment [35]. Additionally, community-based environmental management, robust supply chain management for antimalarial drugs, and a referral system from the community settings to health centers played a role in reducing malaria transmission [36]. Furthermore, the introduction and scaling up of more effective LLINs, particularly those treated with a combination of pyrethroids and piperonyl butoxide (PBO), might have significantly impacted malaria control efforts in Rwanda. Indeed, PBO enhances the efficacy of pyrethroids by inhibiting the enzymes that mosquitoes use to detoxify these insecticides, effectively managing resistance and increasing mosquito mortality. This combination has been shown to maintain effectiveness even in areas with high pyrethroid resistance [37]. Improvements in IRS might have contributed to these declines. The strategic use of new classes of insecticides and the rotation among them have helped overcome the challenges posed by insecticide resistance ensuring the continued effectiveness of IRS in reducing mosquito populations and interrupting malaria transmission [38, 39]. Additionally, innovative vector control tools, such as the use of drones for larvicide application, might have had a positive impact in reducing mosquito larvae. Despite the decline in cases post-2018, the emergence of artemisinin resistance in Rwanda calls for rigorous monitoring and adaptation of treatment protocols [10]. The study results further confirm the positive impact of these interventions, providing evidence that supports the effectiveness of the Rwandan government comprehensive approach in fighting malaria.

Spatially explicit analysis highlighted the heterogeneity of malaria burdens across Rwanda [40]. The high burden in districts like Ngoma, contrasted by significantly lower burdens in districts such as Nyabihu, suggests that local factors like ecologic conditions, vector control practices, healthcare access, and socioeconomic status influence malaria transmission [41]. The cluster analysis was critical in understanding the spatial distribution and demographic segmentation of malaria risk, which is essential for tailored public health interventions. For children under the age of 5 years, significant malaria risk clusters in districts such as Bugesera, Gasabo, and Gisagara call for focused intervention strategies. Females aged above 5 years shared overlapping risk areas with younger children, pointing to similar exposure risks in household and community environments [42].

The Bayesian analysis provided a nuanced understanding of the spatio-temporal dynamics and heterogeneity of malaria risk. Elevated RRs in specific districts over the 11-year study period highlighted the evolving nature of malaria transmission, influenced by ecological, occupational, and behavioral factors and land use patterns [43–45]. The results indicated periods and districts with heightened malaria transmission, emphasizing the need for tailored malaria control strategies based on local risk profiles and demographic vulnerabilities [46].

This analysis does more than just illuminate demographic disparities; it also uncovers district-specific malaria risk. By targeting high-risk populations and adapting interventions to meet the unique needs of these districts, we enhance public health strategy efficiency and effectiveness. This tailored approach maximizes healthcare resource utilisation and responds precisely to the specific demographic and geographic patterns of malaria transmission. Moreover, such strategic alignment addresses the fundamental causes of health inequities, underscoring the importance of improving health equity through focused interventions. Ultimately, this strategy leads to more effective malaria management and reduces disparities across different demographic groups.

This study has important ramifications for Rwanda’s national malaria control program. The findings advocate for a dynamic, adaptive approach to malaria control, integrating socioeconomic, ecological, and behavioural factors. Enhanced surveillance and spatial targeting of interventions, particularly in regions with persistently high RRs, are crucial. Understanding risk profiles across different districts and demographic groups allows for more effective resource allocation and tailored interventions, including the distribution of LLINs, IRS, and community education programs. Additionally, the study highlights the necessity of adaptive management and district-specific strategies, informed by local transmission patterns and demographic vulnerabilities.

The strength of this study lies in its comprehensive approach, utilising advanced statistical methods such as Bayesian spatially explicit models and spatio scan statistics to analyze an 11-year dataset, providing a detailed understanding of the spatio-temporal dynamics of malaria transmission across different demographic groups.

However, the study’s focus on spatio-temporal dynamics does not address the underlying causal pathways. For instance, it has been suggested that climate change might have a broad impact on vector ecology and malaria transmission. In addition, the behavioral aspects of the human population and mosquito vectors, such as changes in human movement patterns or mosquito biting behavior, were not extensively explored. Future research should focus on the long-term sustainability and effectiveness of control measures in the face of changing environmental and demographic conditions and delve deeper into socioeconomic factors influencing malaria prevalence and control effectiveness.

## Conclusions

The current study’s spatio-temporal analysis of malaria in Rwanda from 2012 to 2022 provides new insights into the evolving patterns of malaria transmission, underlining not only the surge in malaria cases from 2014 to 2018, but also the potential underlying factors contributing to these trends. Our findings reveal significant demographic vulnerabilities, particularly among females above 5 years of age and under 5-year-old children, stressing the need for targeted interventions in these groups. The observed increase in malaria cases during this 11-year period can be attributed to a combination of climatic variations, ineffective use of LLINs, resistance of Anopheles gambiae mosquitoes to pyrethroids, and changes in mosquito behavior favoring outdoor biting. Importantly, the study has identified distinct malaria ecozones in Rwanda, suggesting that a one-size-fits-all approach to malaria control is not be feasible. Instead, district-specific strategies considering local ecological, biological, and socio-economic conditions are essential. The decline in malaria cases post-2018, most likely attributed to government-led initiatives such as training CHWs, underscores the potential of localised strategies. Furthermore, the new challenge of artemisinin resistance calls for a robust surveillance system to monitor drug efficacy and vector resistance patterns continually. The persistent high transmission rates in some districts call for a refined focus within the national malaria control program, to address these micro-epidemiological variances effectively.

## Data Availability

All data generated or analyzed during this study are included within the manuscript. Additionally, an interactive web application for further exploration and visualization of the data is available at https://paulamoraga.shinyapps.io/malariarwandaapp/.

## Abbreviations

HMIS: Health management information system
IRS: Indoor residual spraying
LLINs: Long-lasting insecticidal nets
LSM: Larval source management
RR: Relative risk
SDGs: Sustainable development goals
WHO: World Health Organization
